# QGRS-Conserve: a computational method for discovering evolutionarily conserved G-quadruplex motifs

**DOI:** 10.1186/1479-7364-8-8

**Published:** 2014-05-01

**Authors:** Scott Frees, Camille Menendez, Matt Crum, Paramjeet S Bagga

**Affiliations:** 1 Department of Computer Science, Ramapo College of New Jersey, 505 Ramapo Valley Road, Mahwah, NJ 08807, USA; 2 Department of Bioinformatics, Ramapo College of New Jersey, 505 Ramapo Valley Road, Mahwah, NJ 08807, USA

**Keywords:** G-quadruplex, Computational method, *Cis*-regulatory motifs, Evolutionary conservation

## Abstract

**Background:**

Nucleic acids containing guanine tracts can form quadruplex structures via non-Watson-Crick base pairing. Formation of G-quadruplexes is associated with the regulation of important biological functions such as transcription, genetic instability, DNA repair, DNA replication, epigenetic mechanisms, regulation of translation, and alternative splicing. G-quadruplexes play important roles in human diseases and are being considered as targets for a variety of therapies. Identification of functional G-quadruplexes and the study of their overall distribution in genomes and transcriptomes is an important pursuit. Traditional computational methods map sequence motifs capable of forming G-quadruplexes but have difficulty in distinguishing motifs that occur by chance from ones which fold into G-quadruplexes.

**Results:**

We present Quadruplex forming ‘G’-rich sequences (QGRS)-Conserve, a computational method for calculating motif conservation across exomes and supports filtering to provide researchers with more precise methods of studying G-quadruplex distribution patterns. Our method quantitatively evaluates conservation between quadruplexes found in homologous nucleotide sequences based on several motif structural characteristics. QGRS-Conserve also efficiently manages overlapping G-quadruplex sequences such that the resulting datasets can be analyzed effectively.

**Conclusions:**

We have applied QGRS-Conserve to identify a large number of G-quadruplex motifs in the human exome conserved across several mammalian and non-mammalian species. We have successfully identified multiple homologs of many previously published G-quadruplexes that play post-transcriptional regulatory roles in human genes. Preliminary large-scale analysis identified many homologous G-quadruplexes in the 5′- and 3′-untranslated regions of mammalian species. An expectedly smaller set of G-quadruplex motifs was found to be conserved across larger phylogenetic distances. QGRS-Conserve provides means to build datasets that can be filtered and categorized in a variety of biological dimensions for more targeted studies in order to better understand the roles that G-quadruplexes play.

## Background

**Figure 1:**
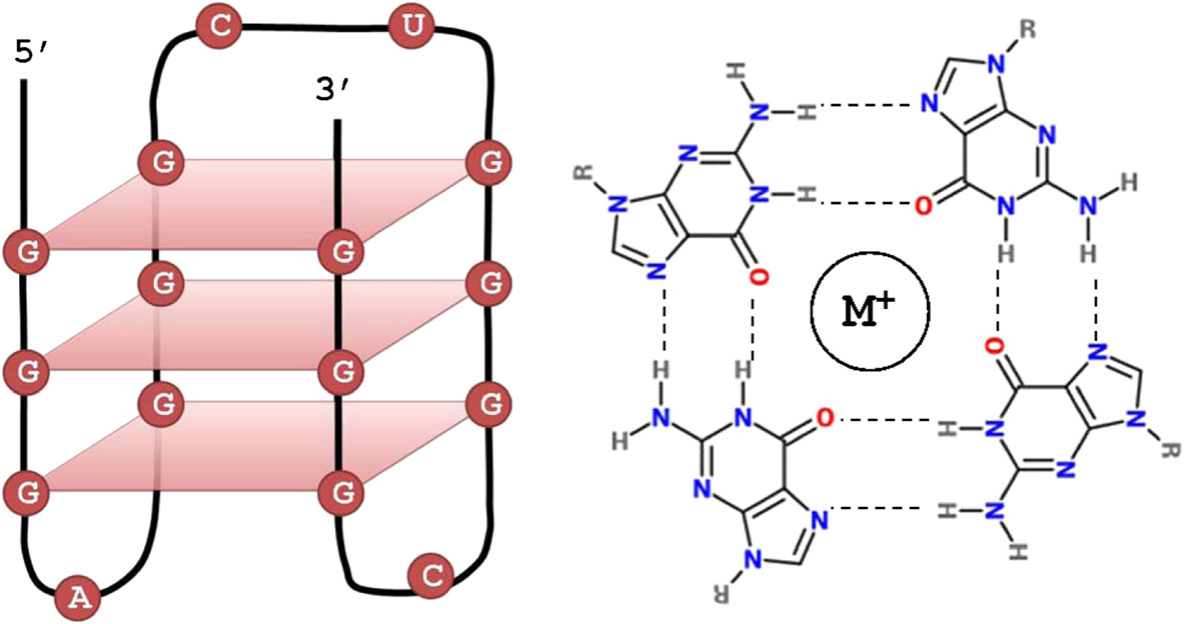
**G-quadruplex structure.** Left: an intramolecular G-quadruplex formed by a **G**_3_A**G**_3_CU**G**_3_C**G**_3_ RNA motif. Right: a G-tetrad structure.

Nucleic acids containing guanine tracts can form quadruplex structures via non-Watson-Crick base pairing [1,2]. These structures, known as G-quadruplexes, are composed of stacked G-tetrads - square co-planar arrays of four guanine bases each (Figure 1). Each tetrad in the G-quadruplex is stabilized by cyclic Hoogsteen hydrogen bonding between the four guanines [3-6]. G-quadruplexes can be formed by unimolecular interactions via repeated folding of a single nucleic acid molecule or through multimolecular interactions between two or four strands. Our work focuses on unimolecular G-quadruplex formation which has been the target of most genomic studies and is more likely to be encountered in physiological conditions [6,7].

Formation of G-quadruplexes in DNA and RNA is associated with regulation of important biological functions [8]. There have been several reports of G-quadruplex involvement in a variety of human diseases [9-11]. Consequently, G-quadruplexes are being considered as targets for therapeutic purposes [9,12,13]. DNA G-quadruplexes can influence transcription, telomere biology, genomic rearrangements and instability, DNA recombination, DNA repair, DNA replication, and epigenetic mechanisms [14-18]. RNA G-quadruplexes readily form *in vivo*[19] and are considered more stable than DNA G-quadruplexes [20,21].

G-quadruplexes have been reported in untranslated as well as translated regions of mRNAs [22,23] and have been shown to function as *cis-*regulatory elements of translation and alternative splicing [24-29]. For example, G-quadruplex structures located in the 5′-UTR (untranslated region) of human fibroblast growth factor 2 (FGF2) [30] and human vascular endothelial growth factor (VEGF) [25] mRNAs act as internal ribosomal entry sites (IRES) for cap-independent translation initiation. Deletion of a conserved G-quadruplex motif in the 5′-UTR of MECP2 gene has been associated with reduced protein product and autism, suggesting that a G-quadruplex plays a role in normal protein synthesis [11]. Formation of G-quadruplexes in this region can also inhibit translation of neuroblastoma Ras (NRAS) oncogene [24] and ADAM10 - responsible for anti-amyloidogenic processing of the amyloid precursor protein (APP) [31]. G-rich sequences capable of forming G-quadruplexes in the 3′-UTR have been shown to enhance cleavage and polyadenylation of mammalian pre-mRNAs [32-35], influence RNA turnover [29], and to play a role in subcellular mRNA sorting [36].

G-quadruplexes can be detected in the lab using UV spectroscopy and fluorescent tags [37,38]. Structure-specific antibodies can be used to quantitatively visualize DNA G-quadruplexes in human cells [39,40]. However, for large-scale analysis, researchers turn to computational approaches for identifying regions likely to form G-quadruplexes. Bioinformatics studies have been conducted to map potential G-quadruplex motifs in genomic regions [41-46], splice regions [47], and untranslated regions [48] of mammals, and genomes of yeast [49] and prokaryotes [50]. Most computational algorithms rely on identifying sequence motifs capable of forming G-quadruplex structures. These algorithms primarily analyze G-tract lengths and loop lengths in the nucleotide sequences.

In the cell, many factors - such as the presence of a cation within or between co-planar guanines - may contribute to G-quadruplex formation. Furthermore, G-quadruplex formation can be transient - they may form and dissolve under physiological conditions intermittently. These complexities can make computational mappings subject to false-positives, where motifs occurring by chance will be identified along with motifs representing real G-quadruplexes with significant biological impact.

One way to reduce the likelihood of false-positives when predicting G-quadruplex formation is by examining the motif's conservation across species. Nucleotides which are integral to G-quadruplex formation are known to be conserved and less likely to be polymorphic [51]. This increased level of conservation has been specifically observed in *Saccharomyces*[52]. Capra and colleagues measured this conservation in two ways: at the motif level and at the nucleotide level. Motif-level conservation was defined as a binary result based on whether an overlapping motif was found in two aligned sequences. Nucleotide conservation was calculated by comparing the number of conserved bases within a motif against conserved nucleotides within the neighboring 100 nt.

In this paper we present quadruplex forming G-rich sequences (QGRS)-Conserve, a strategy for identifying highly conserved G-quadruplex motifs in homologous nucleotide sequences. Our approach differs from Capra and co-workers in that it evaluates not only location conservation but also the conservation of structural features of the G-quadruplex motif - such as loop lengths, number of tetrads, and the total length of the structure. We also present strategies for dealing with specific challenges relating to overlapping G-quadruplex motifs and the impact they have on conservation analysis. Our technique allows for the filtering of motifs based on qualitative conservation and promotes accurate wide-scale analysis of G-quadruplexes within exomes, transcriptomes, and genomes. Since conservation suggests biological relevance, our tools can help researchers focus on motifs likely to be of high scientific interest. We present the summative results from a database of G-quadruplexes created using the QGRS-Conserve.

## Methods

QGRS-Conserve is a computational method for calculating motif conservation across exomes that supports filtering to provide researchers with more precise methods of studying distributions patterns. The work presented in this paper focuses on G-quadruplexes found in mRNA; however, the algorithms and principles could be similarly applied when working with DNA.

### Identifying G-quadruplexes

QGRS-Conserve identifies QGRS (predicted G-quadruplexes) in nucleotide sequences based on algorithms described previously [53,54]. Putative G-quadruplexes are identified using the motif G_*x*_N_*y*1_G_*x*_N_*y*2_G_*x*_N_*y*3_G_*x*_. The motif consists of four guanine tracts of equal size interspersed by three loops. In this expression, *x* represents the number of guanine tetrads, and *y*1–*y*3 represent the loops (gaps). The size of each G-tract corresponds to the number of stacked G-tetrads forming the quadruplex structure (Figure 1).

This motif expression is consistent with those in other published methods, although some methods place differing restrictions on the number of tetrads and/or loop lengths applied [55,56].

The numeric quantification of predicted stability is useful in facilitating filtering and searching across large datasets. To quantify stability, we calculate ‘G-scores’ based on a previously published strategy [53,54] for each identified QGRS. While this stability is not used in our conservation measurements, it can be used to filter which QGRS that are ultimately used for conservation calculations.

### Measuring conservation

Finding a QGRS motif - especially one that yields a high G-score - is of interest. However, finding QGRS that appear to be under evolutionary constraint - and hence likely to play important biological roles - is our larger goal. Measuring a QGRS's conservation across homologous genes is a way of further measuring, indirectly, the likelihood that the QGRS does indeed form a G-quadruplex. The combination of evolutionary conservation and high G-scores can be a strong indicator that the nucleotide region in question requires more direct study.

QGRS-Conserve measures both location and structural conservation of QGRS, using four numerical components: (1) their location (overlap) within aligned sequences, (2) tetrad similarity, (3) loop length similarity, and (4) total QGRS length similarity. For each pairing of QGRS between two homologs, a weighted score between 0 and 1 is computed - where 1 indicates high conservation. We will refer to this score as the pair s ‘conservation score.’

### Location conservation

Aligned homologous nucleotide sequences are expected to contain significant conserved stretches, representing evolutionarily conserved function. It is therefore reasonable to assume that QGRS motifs in the same or similar location across aligned nucleotide sequence homologs have undergone similar evolutionary pressure and can be assigned with homologous status. To evaluate how well the two QGRS motifs align, a semi-global alignment between homologs is performed; the *principal* nucleotide sequence is typically from *Homo sapiens* and the *comparison* sequence is from another species. A semi-global alignment is preferred over the full global alignment because of the variation between the lengths of untranslated regions across orthologous mRNAs.

Sixty-five percent of our *conservation score* is dedicated to the overall location similarity between the two QGRS. Once a set of homologous sequences has been aligned, each pair of QGRS is assigned an *overlap score*. The overlap score, like the overall conservation score, ranges from 0.0 to 1.0. The premise is that pairs that do not occupy the same relative region should have low scores approaching 0, while pairs highly proximate (while not necessarily precisely aligned) receive scores nearing 1.0.

**Figure 2:**
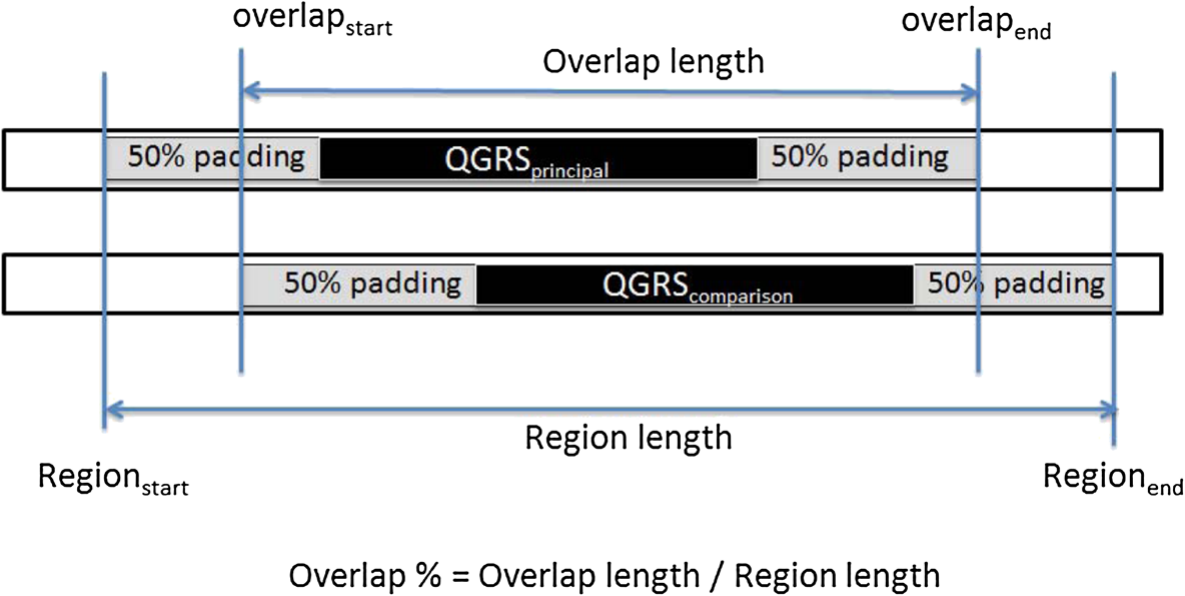
**Overlap percentage computation.** The extension of sequence length using 50% of the total sequence length as padding inflates overlap scores for proximate sequences.

To compute the overlap score for a given QGRS pair, a start and ending nucleotide position is determined for the QGRS in the principal and comparison sequences. Recognizing that up/downstream sequence variations can easily cause QGRS pairs to be misaligned by several nucleotides, we apply padding to each start and end point of the QGRS of 50% the total length of the smaller QGRS. The percentage overlap between the modified endpoints is computed as shown in Figure 2. Note that padding prevents the outright elimination of proximate but non-overlapping QGRS.

Any pairing with an overlap score greater than 0 is transformed through a step function to eliminate the penalty of slightly misaligned motifs. Overlap percentages of 85% or greater is given a score of 1.0, and all other scores are assigned an interpolation between 0 and 1.

### Motif structural conservation

An additional 20% of the conservation score is based on similarity in the number of tetrads (length of G-tracts). The number of tetrads can determine stability, as well as regulatory characteristics of G-quadruplexes [57]. Each QGRS has a number of tetrads ranging from 2 to *N*, and the percentage similarity among the two is determined by dividing the smaller over the larger. This initial ratio is transformed by a step function to assign a numeric score, where any ratio at or below 50% is assigned a 0 and between 50% and 100% is mapped between 0.5 and 1.0. For example, two QGRS of three and four tetrads would have a similarity score of 0.75, where two with two and four tetrads would be assigned a score of 0.

Since the number of tetrads tends to be quite small (most QGRS have tetrad counts less than 5), we have also accounted for fractional differences. Rather than considering QGRS pairs with two and three tetrads to have a difference of a full tetrad, we consider the individual tracts within the motif with shorter G-tracts to examine how close it is to becoming a full three-tetrad QGRS. For example, the QGRS **GGG**ACGTTC**GG**ATT**GGG**TTACCA**GGG** would be considered (only for the purposes of the tetrad similarity computation) to have a tetrad length of 2.75 - as three of the four tracts have an adjacent extra guanine, meaning that a single mutation in the other tract would have yielded a three-tetrad QGRS.

This method creates a more favorable tetrad similarity score, and therefore a higher conservation score, and more accurately measures the similarity of G-quadruplex structures putatively formed by QGRS motifs in question.

Similarity among loop lengths is also factored into the conservation score calculation, accounting for 10% of the overall score. The loop length similarity is taken as the average percentage similarity of each of the three loops. Each loop's similarity is simply the smaller loop length divided by the larger, and a similar step function is applied to the score to transform any percentage less than 50 to 0.

The last 5% of the conservation score is the overall length score. If there is 60% overall length similarity or lower, a length score of 0 is assigned. Larger percentages are interpolated to scale along a range from 0 to 1. The overall length score only accounts for 5% because the parameter of the overall length is already somewhat incorporated in the tetrad and loop length similarity scores but is still important to consider and record as it can be used to filter data as needed.

Three of the four numerical components are based on structural determinants of G-quadruplex formation. For these components, a relatively higher score (20%) was chosen for tetrad similarity among structural components because the number of tetrads can determine stability as well as the regulatory characteristics of G-quadruplexes [57]. A fair evaluation of conservation between a QGRS pair should, therefore, involve a close comparison of the number of tetrads.

Since the location of G-quadruplex motifs in the mRNAs is of high biological relevance, a 65% weighting was assigned to location/overlap numerical component of conservation score.

We have developed our quantitative scoring methods and component weightings in an effort to generally reflect the current state of awareness in the G-quadruplex field. It is an active area of research and to the best of our knowledge, ours is one of the first attempts to quantify G-quadruplex motif conservation.

It should be noted that other factors observed in literature have not been incorporated into our conservation score, including loop composition similarity and ion binding preference similarity of G-quadruplexes [58-60]. These characteristics of G-quadruplex structures are still being actively researched. Until a clear picture emerges, we felt that it would not be possible to incorporate these factors into the conservation score at present time.

### Implementation

**Figure 3:**
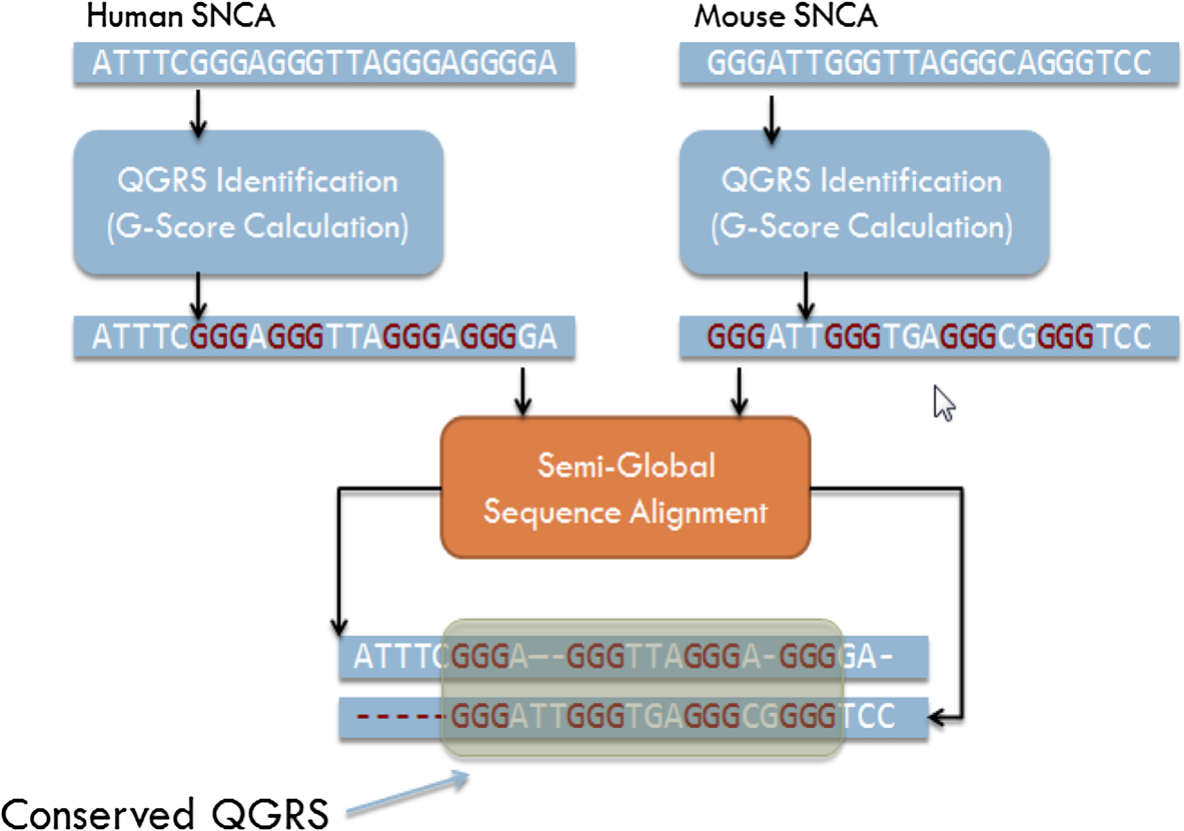
**Algorithm stages for SNCA mRNA.** Overview of the algorithm stages for QGRS identification (independently) on each mRNA, performing a semi-global alignment on the homologs, and finally evaluating the QGRS pair for conservation.

QGRS-Conserve is implemented as a series of three stages (Figure 3). Once the full sequence data for the principal and comparison genes have been obtained, the first stage is to identify all QGRS within each sequence independently.

(1) $$Q_{p}=\left\{\text{set}\;\mathrm{of}\;\text{all}\;\text{QGRS}\;\text{found}\;\text{within}\;\text{principal}\;\text{mRNA}\right\}$$

(2) $$Q_{c}=\left\{\text{set}\;\mathrm{of}\;\text{all}\;\text{QGRS}\;\text{found}\;\text{within}\;\text{comparison}\;\text{mRNA}\right\}$$

The second stage is a semi-global alignment of the two sequences. A semi-global alignment is performed without consideration of the location of the QGRS found in the first stage and produces an optimal alignment over the entire sequence - avoiding penalizing the gaps at the ends of the strands. The semi-global alignment typically injects gaps into each sequence representing differences with the aligned sequence. Care is taken within the program to preserve a record of the location of each QGRS found in the first stage both before and after these gaps are introduced. Our program uses the Needleman-Wunsch Semi-Global alignment web service provided by EMBOSS, which can be accessed at (http://www.ebi.ac.uk/Tools/webservices/services/psa/emboss_needle_soap).

The final stage is the calculation of the conservation score. Here, each QGRS in *Q*_p_ is paired with each QGRS in *Q*_c_. Many of these pairings are extraneous, they represent QGRS that maybe in very different locations within their respective genes and are not necessarily homologous - the conservation score computation will automatically dispose of low scoring pairs, as their overlap score will be 0. For each pair, the composite conservation score consisting of scores for overlap (using nucleotide positions after the gaps from alignment are added), loop length similarity, tetrad similarity, and overall length similarity is computed. Loop length and overall length similarities are based on nucleotide counts *without* the gaps resulting from alignment.

### Efficient large-scale analysis

The QGRS-Conserve algorithm has been made available through a web application previously called QGRS Predictor [61], the functionality of which has been published elsewhere [62]. The interactive web interface allows a user to examine a single pair of mRNA sequences at a time, which is useful when researchers already know which set of homologs they are interested in studying. The broader goal of our research is to study the distribution patterns of conserved QGRS *across entire exomes/transcriptomes* for better understanding the biological roles of G-quadruplex motifs in post-transcriptional regulation of gene expression.

We have created a database of conserved QGRS using QGRS-Conserve in conjunction with listings of homologous mRNA pairings available through NCBI's Homologene database [63]. At the time of writing, our master database contains conservation computations of the entire human exome compared against homologs within the exomes of *Mus musculus*, *Pan troglodytes*, *Canis lupus familiaris*, *Danio rerio*, *Caenorhabditis elegans*, and *Kluyveromyces lactis*. Large-scale analysis presents many challenges beyond the interactive use-case, and we now describe additional solutions we have developed specifically to allow efficient storage and analyses of QGRS across entire exomes.

### Overlapping QGRS

**Figure 4:**
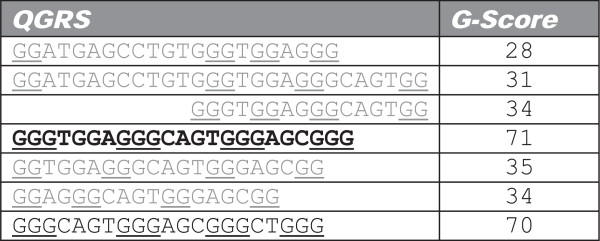
**Overlapping QGRS yielding different G-scores.** A small sample of hundreds of overlapping QGRS found in the ‘GGATGAGCCTGTGGGTGGAGGGCAGTGGAGCGGGCTGGG’ sequence of human GREB1 gene.

QGRS within a sequence are said to overlap when their positions (start to end) in the nucleotide sequence overlap (Figure 4). Overlapping QGRS may differ in total length, loop size, or loop sequence; any of them have the potential to form intramolecular quadruplexes in the cell, many of which may be stable. Several strategies for dealing with overlapping QGRS can be found in the literature. One approach is to focus on *regions* where G-quadruplexes may be formed, rather than specific motifs. These regions, which contain overlapping and proximate G-quadruplexes, are called regions with ‘G4 potential,’ or G4P [43]. If the presentation of specific motifs is desired (such as when evaluating structural conservation), overlapping motifs may be preserved [41] or they may be filtered based on the selection of the motif with the highest number of G-tracts, the higher predicted stability [54], or other characteristics. Typically, this filtering is acceptable, as the tools merely highlight regions in a sequence where G-quadruplexes are likely to be found; they do not claim the specific motif presented will be the one and only G-quadruplex formed among the overlapping motifs.

When presenting the analysis between two mRNA sequences in the interactive interface, all pairings of QGRS across the species are considered, and all homologous pairs meeting the user's specified filter criteria (i.e., minimum conservation score) are presented. This listing *includes* the pairing of overlapping QGRS. We chose this approach because when performing analysis to measure conservation across species, the removal of overlapping QGRS cannot be done without impact on results. When highlighting conservation, we not only consider the characteristics of an individual QGRS but also how similar it is with QGRS in the comparison species sequence. Due to this complication, it is not optimal to remove overlaps until conservation computations across all QGRS pairs can be computed.

### Overlapping QGRS and conservation calculations

**Figure 5:**
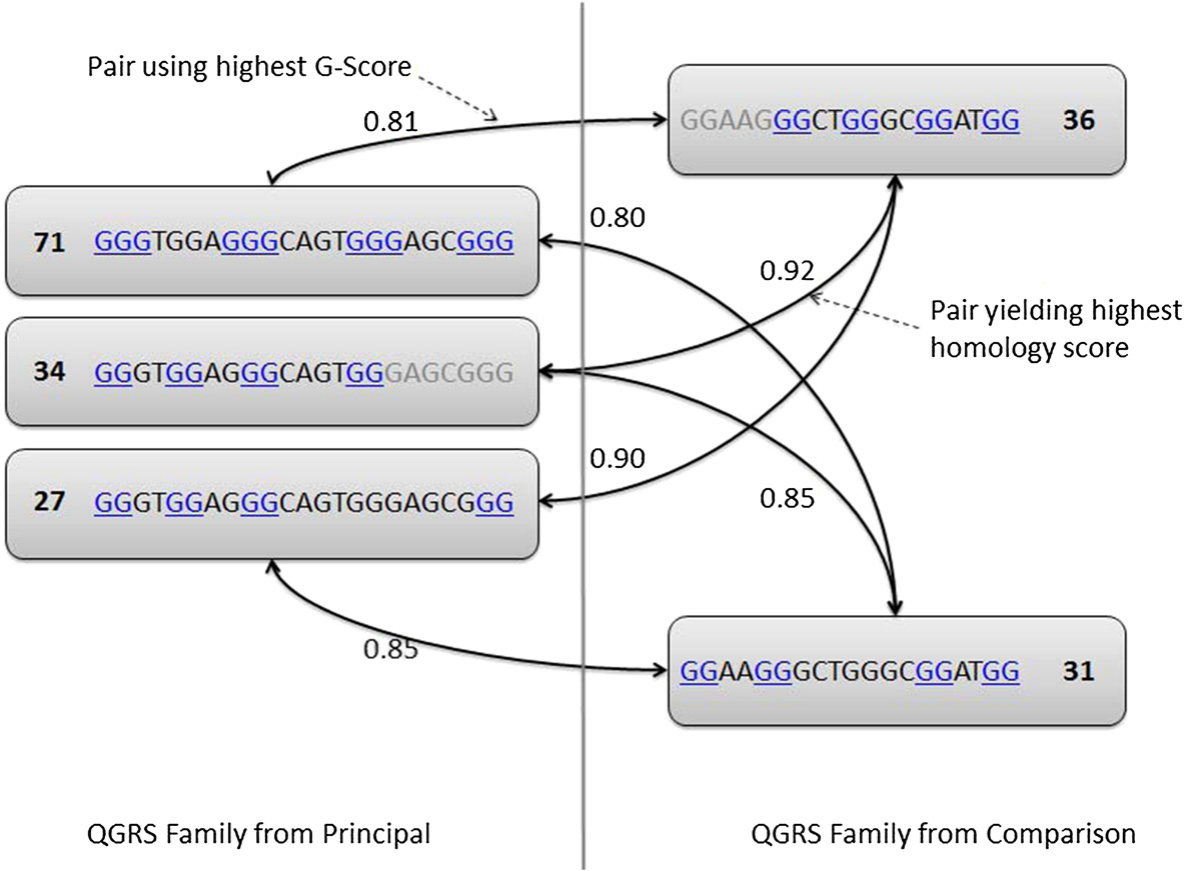
**Choosing QGRS considered for conservation calculations.** The number next to each QGRS motif represents its calculated G-score. This figure demonstrates how it is possible that QGRS with highest G-score do not necessarily yield the highest conservation score.

Unlike when performing an interactive analysis, in large-scale analysis, the consideration of all overlapping pairings of QGRS is impractical and ineffective. To illustrate the challenge of dealing with overlapping QGRS when computing conservation scores, consider a situation where multiple overlapping QGRS are found in similar areas of two aligned mRNA sequences [principal and comparison (homologous) sequences] as shown in Figure 5. Each QGRS found in the principal sequence have their own characteristics (such as number of G-tracts and loop lengths), yielding a different G-score (integer to the left of the nucleotide sequence).

We have adopted the term ‘QGRS family’ to represent a set of overlapping QGRS within a sequence. When choosing which QGRS within a family to compare with QGRS in the homologous sequence, we could choose the one with the highest predicted stability (G-score); however, optimizing on predicted stability *first* may not yield the highest conservation score present among QGRS families. It is quite possible that comparing QGRS instances with slightly suboptimal predicted stability (i.e., relatively lower G-scores) may yield better similarity. As an example, in Figure 5, selecting the QGRS instance within each family with the highest G-score might yield a conservation score of 0.81, where the optimal score across the two families might be far larger (0.92 in this case).

Comparing only QGRS with the highest G-Score can potentially underestimate conservation. However, computing and storing *all* combinations of overlapping motif present serious problems when conducting any large-scale analysis, not only for performance reason but because the presence of overlapping pairs can skew aggregate statistics concerning the numbers of QGRS found in particular regions of the sequences.

### Filtering overlapping motifs

To address overlapping motifs, we used a compromise that allows us to report the highest conservation measurement across families of overlapping motifs while *maintaining* only a single specific representative motif from each family. The solution has the characteristic of identifying representative QGRS within specific mRNA independently from homologs it is compared against - allowing for the cross-referencing of the same QGRS in one gene (e.g., from human) against many homologs from other species.

#### Step 1: locating QGRS instances in each mRNA

For a given pair of mRNA (principal and comparison), the first stage of processing simply identifies all QGRS - including overlapped motifs, giving us two sets of QGRS.

(3) $$\begin{array}{ll} Q_{p}= & \left\{\text{set}\;\mathrm{of}\;\text{all}\;\text{QGRS}\;\text{found}\;\text{within}\;\text{principal}\;\text{mRNA},\right)\\ & \left(\text{including}\;\text{overlaps}\right\} \end{array}$$

(4) $$\begin{array}{ll} Q_{c}= & \left\{\text{set}\;\mathrm{of}\;\text{all}\;\text{QGRS}\;\text{found}\;\text{within}\;\text{comparison}\;\text{mRNA},\right)\\ & \left(\mathrm{including}\;\mathrm{overlaps}\right\} \end{array}$$

#### Step 2: group QGRS into overlapping families

For **Q**_p_ and **Q**_c_, the QGRS are grouped into a collection of *QGRS families*, where each QGRS within a given family differ in nucleotide position by less than five bases with all other QGRS in the family. Note that it is possible (while not necessarily common) for QGRS to fall into multiple families. This provides us with two sets of families:

(5) $$\begin{array}{ll} F_{p}= & \left\{\text{subsets}\;{\text{ofQ}}_{p}\text{where}\;\text{each}\;\text{QGRS}\;\mathrm{in}\;a\;\text{given}\;\text{family}\;\right)\\ & \left(\text{overlap}\right\} \end{array}$$

(6) $$\begin{array}{ll} F_{c}= & \left\{\text{subsets}\;{\text{ofQ}}_{c}\text{where}\;\text{each}\;\text{QGRS}\;\mathrm{in}\;a\;\text{given}\;\text{family}\;\right)\\ & \left(\text{overlap}\right\} \end{array}$$

#### Step 3: persisting QGRS instances to the database

A QGRS record is created for each family in **F**_p_ and **F**_c_. The QGRS record's identifier in the database will correspond to the family number (ordered from 5′ to 3′ by starting nucleotide position). The structural characteristics (G-tracts, loop lengths, etc.) and G-score will be saved corresponding to the QGRS within the family with the highest G-score. Note while only one QGRS per family is stored in the database as a QGRS record, the set of all QGRS found in the family are not discarded immediately - they are utilized in step 5.

#### Step 4: pair families in F_p_ and F_c_ for conservation computation

Each QGRS family in **F**_p_ is paired with each family in **F**_c_, yielding another set **X**_**f**_, where each element in **X**_f_ is a *pair* of QGRS families (one from principal and one from comparison mRNA).

(7) $$X_{f}=\left\{\text{cross}\;\text{product}\;\mathrm{of}\;F_{p}\;\text{and}\;F_{c}\right\}$$

For each pair of QGRS families in **X**_f_, a conservation record will be created in the database. A conservation record will contain the identifier of the QGRS from the principal and comparison mRNA. In this case, the conservation record will reference the representatives selected in step 3.

#### Step 5: conservation computations

In order to compute the conservation between two QGRS families, a new set of pairings, **X**_q_, is computed. **X**_q_ represents each QGRS instance from the principal family paired with each from the comparison family.

(8) $$\begin{array}{ll} X_{q}= & \left\{\text{cross}\;\text{product}\;\mathrm{of}\;\text{QGRS}\;\text{instances}\;\mathrm{in}\;\text{family}\;\text{from}\;\right)\\ & \left(\text{principal}\;\text{with}\;\text{family}\;\text{from}\;\text{comparison}\right\} \end{array}$$

The highest conservation score (calculated using the procedure described in previous sections) in **X**_q_ for each pair of families in **X**_f_ is then persisted to the database.

Our filtering design has several implications. Firstly, all possible QGRS pairs are evaluated across a set of homologous mRNA, not only the ones with the highest G-score among them. This prevents the analysis from missing highly conserved motifs that may warrant special investigation. Secondly, individual QGRS recorded for a particular mRNA correspond to the QGRS within each family with the highest G-score. This means the set of actual QGRS stored for a particular mRNA is independent from conservation scores across homologs. Since the same QGRS represent a single family of overlapping QGRS regardless of which homolog it is being compared with, it is possible to cross-reference QGRS in a single mRNA with potentially dozens of homologs. This feature allows for queries such as ‘find all QGRS in the human SNCA gene which have highly homologous representations in three or more other homologs from other species.’

## Results and discussion

The algorithms and methods behind QGRS-Conserve allow for efficient identification of conserved QGRS across large sets of nucleotide sequences and include strategies to handle overlapping QGRS without sacrificing the fidelity of the conservation scoring. These strategies are critical to make large-scale analysis efficient and tractable.

We have used QGRS-Conserve to create a database of QGRS records and corresponding conservation scores focused on human mRNA compared against mRNA homologs from seven species. At the time of writing, over 68,000 mRNA homolog pairs have been analyzed, yielding over 433,000 computationally mapped QGRS instances in the human exome alone and over 1 million across all seven species. This dataset includes information about 343,153 human QGRS (in 16,684 human mRNAs) conserved in at least one ortholog. Conservation (conservation score > 0.95) of human QGRS was calculated against homologs in seven species: *M musculus*, *P troglodytes*, *C. lupus familiaris*, *D rerio*, *C. elegans*, and *K. lactis*.

We have collected information for the categorization of QGRS by tetrads, loop lengths, G-score, conservation, absolute location (both nucleotide positions, and regional - 5′-UTR, CDS, 3′-UTR), relative location (e.g. distance from polyA signals/sites), mRNA similarity (alignment scores), and gene ontology terms.

We have created a web interface, open to the public, which allows researchers to browse the database. This site can be found at http://qdb.ramapo.edu. The data is fully searchable and allows the user to obtain listings of QGRS filtered by mRNA sequence, associated gene ontology, stability (G-score), number of tetrads, and conservation score. As we continue to incorporate more species and search functionality, such features will be added to the web site.

Table 1 shows an overview of our database and highlights the ability to filter out potential false-positives at the motif level using conservation. When considering conservation between human and other species such as dog and mouse, conservation scores filtered at 0.95 or above eliminate over 50% of the mapped human QGRS. When coupled with region considerations (for example, only considering 5′ or 3′-UTR), this computation allows researchers to locate QGRS of high potential interest quite quickly. The following sections discuss the categorizations and trends in Table 1 in more detail.

**Table 1:** An overview of human QGRS conservation across seven orthologous exomes

**Homologous exome**	**QGRS distribution**		
	**Tetrads**		
	**2**	**3**	**4+**
*Pan troglodytes* (14317)			
Entire mRNA	255453 (75%)	16731 (68%)	612 (61%)
5′-UTR	28398 (65%)	3512 (58%)	165 (53%)
CDS	170536 (78%)	6887 (75%)	195 (75%)
3′-UTR	61244 (72%)	6747 (68%)	260 (59%)
*Canis lupus familiaris* (14513)			
Entire mRNA	132440 (36%)	5121 (19%)	144 (13%)
5′-UTR	6249 (14%)	635 (10%)	25 (7%)
CDS	121915 (52%)	3744 (38%)	91 (33%)
3′-UTR	5996 (7%)	858 (8%)	33 (7%)
*Mus musculus* (15231)			
Entire mRNA	156152 (41%)	6461 (23%)	200 (17%)
5′-UTR	13600 (29%)	1367 (20%)	63 (17%)
CDS	128755 (52%)	3238 (31%)	75 (25%)
3′-UTR	16543 (18%)	2010 (18%)	67 (12%)
*Danio rerio* (10963)			
Entire mRNA	29001 (10%)	198 (1%)	(0%)
5′-UTR	391 (1%)	6 (<1%)	(0%)
CDS	28580 (16%)	191 (3%)	(0%)
3′-UTR	188 (<1%)	4 (<1%)	(0%)
*Drosophila melanogaster* (4407)			
Entire mRNA	5970 (6%)	29 (<1%)	(0%)
5′-UTR	153 (1%)	1 (<1%)	(0%)
CDS	5798 (9%)	26 (1%)	(0%)
3′-UTR	42 (<1%)	2 (<1%)	(0%)
*Caenorhabditis elegans* (3035)			
Entire mRNA	1543 (2%)	(0%)	(0%)
5′-UTR	13 (<1%)	(0%)	(0%)
CDS	1534 (4%)	(0%)	(0%)
3′-UTR	5 (<1%)	(0%)	(0%)
*Kluyveromyces lactis* NRRL Y-1140 (1298)			
Entire mRNA	444 (2%)	(0%)	(0%)
5′-UTR	1 (<1%)	(0%)	(0%)
CDS	443 (3%)	(0%)	(0%)
3′-UTR	(0%)	(0%)	(0%)

Human QGRS are conserved in untranslated as well as translated regions of orthologous mRNAs. The conservation is more prevalent in mammalian orthologs. Values in *parentheses* in the first column represent the number of human mRNAs with an established ortholog in the given species. Values under ‘QGRS distribution’ represent data for QGRS types with two tetrads, three tetrads, or four and higher number of tetrads. For each tetrad column, the first number represents the number of QGRS in *H. sapiens* mRNA conserved within the given species exome. Data for only QGRS with conservation score ≥0.95 are presented. The second number in parenthesis is the *percentage* of *H. sapiens* QGRS conserved in the given species.

### Categorizing conservation

Table 1 shows the percentage of QGRS found in *H. sapiens* mRNAs where homologous QGRS were found in another organism. Human QGRS were found to be conserved in untranslated as well as translated regions of orthologous mRNAs. Predicted homologous QGRS with two tetrads were expectedly more prevalent than the corresponding motifs with three tetrads, which in turn were identified with higher frequency than QGRS with four or more tetrads. The rate of conservation is highest within the CDS region, across all species examined, in line with the fact that the CDS tends to be more highly conserved as compared to the 5′-UTR and 3′-UTR regions. The untranslated mRNA regions are known to be more variable. Conserved regions in the UTRs are likely to represent regulatory motifs. Therefore, while fewer in number, the identified homologous QGRS in these otherwise less conserved regions are of particular interest for future exploration.

### Conservation among phylogenetic groups

We observed that the rate of QGRS conservation generally increases as the phylogenetic distance from *H. sapiens* decreases. Our analysis noted significant conservation of human QGRS among mammalian species (Table 1), including conservation in the 5′- and 3′-UTR regions. In contrast, almost all of the observed QGRS conservation in non-mammalian organisms could be attributed to the CDS. Non-homeothermic organisms had significantly lower level of conservation for three-tetrad QGRS among the only two species (*D. rerio* and *Drosophila melangoaster*) in which they were detected. In general, there is a distinct lack of conserved human G-quadruplex motifs in the untranslated exomes of the lower organisms. Based on overall sequence variation between such species and *H. sapiens*, these results are expected.

### Conservation of established G-quadruplexes

Conservation is, of course, not a prerequisite for QGRS to actually form a G-quadruplex in a living cell - it is simply a strong clue. To evaluate the potential for our algorithm to provide false negatives (where low conservation scores are assigned to actual G-quadruplexes, such that they may be filtered out during a search), we investigated how our procedure worked with known G-quadruplexes reported in the literature.

We targeted ten previously published human G-quadruplexes reported for their post-transcriptional regulatory roles in different genes. These genes are involved in a wide variety of important biological processes and diseases (Table 2). G-quadruplexes mapped to 5′- or 3′-UTRs in these genes have been reported for their involvement in regulating 3′-end RNA processing [64,65], translation [11,24,31,66-69], or subcellular localization [36]. Table 2 shows human QGRS mapped to orthologous *M. musculus* genes along with their corresponding QGRS-conservation scores. While similar mappings can be made with other species, we chose *M. musculus* due to its reasonable phylogenetic distance from humans (i.e., data from chimpanzee has extremely high nucleotide conservation overall, while very little conservation of any kind can be found in distant organisms such as fruit flies). Of these ten instances, six were found to have highly homologous QGRS in mouse orthologous genes - meaning using only computational means our tool accurately highlights QGRS already known to form G-quadruplexes. Three of the G-quadruplexes in the human genes could not immediately be matched to QGRS in mouse because the latter were found only in the untranscribed region immediately upstream of the mouse transcription start site (TSS). 5′-UTRs of the currently available mouse NRAS, ADAM10, and YY1 mRNA sequences are shorter than their corresponding established human orthologs (Table 2). To test our method for these G-quadruplexes, we manually entered the upstream data taken from corresponding genomic sequence sources. Using our method with this adjusted sequence data, we found highly homologous QGRS. The homology scores marked with double asterisk in the table are each derived from calculations using sequence data mapped in the upstream genomic sequence prepended to the mRNA sequence of the mouse homolog.

**Table 2:** Mapping QGRS orthologs of previously published human G-quadruplexes

***Homo sapiens***						***Mus musculus***		
**Gene name**	**Biological relevance**	**mRNA (Ref seq)**	**QGRS location**	**QGRS sequence motif**	**Reference**	**mRNA (Ref seq)**	**mRNA similarity with human**^**a **^**(%)**	**QGRS conservation score**
LRP5	Receptor-mediated endocytosis	NM_002335.2	3′-UTR (+136)	GGGGTGGGCAGGGCTGGG	Beaudoin et al. 2013 [64]	NM_008513.3	86	1.00
PIM1	Cancer and apoptosis	NM_002648.3	3′-UTR (+279)	GGGGTGGGGGGTGGGGGTGGG	Arora and Suess 2011 [66]	NM_008842.3	52	1.00
IGF2	Wilms' tumor	NM_001127598.1	3′-UTR (+2194)	GGGGTGGGTGGGGGGCAGTGGGGGCTGGGCGGGG	Christiansen et al. 1994 [65]	NM_010514.3	56	1.00
PSD95	Synaptic junction formation	NM_001365.3	3′-UTR (+685)	GGGAGGGAGGGTGGG	Subramanian et al. 2011 [36]	NM_001109752.1	64	1.00
KISS1	Metastasis suppression	NM_002256.3	3′-UTR (+53)	GGGGCGGGGGCGGGGGGCGGGGACGTAGGGCTAAGGGAGGGG	Huijbregts et al. 2012 [67]	NM_178260.3	49	0.706^b^
NRAS	Rectal cancer	NM_002524.4	5′-UTR (-240)	GGGAGGGGCGGGTCTGGG	Kumari et al. 2007 [24]	NM_010937.2	64	1.00^c^
ADAM10	Anti-amyloidogenic activity	NM_001110.2	5′-UTR (-78)	GGGGACGGGTATGGGCGGG	Lammich et al. 2011 [31]	NM_007399.3	71	1.00^c^
MECP2	Rett syndrome/autism	NM_004992.3	5′-UTR (-108)	GGAGGAGGAGGAGGCGAGG	Bagga and D′Antonio 2013 [11]	NM_001081979.1	74	1.00
YY1	Transcription	NM_003403.3	5′-UTR (-203)	GGGCGCGGGCGCACCGAGGCGAGGGAGGCGGG	Huang et al. 2011 [68]	NM_009537.3	89	0.944^c^
TERF2	Telomeric stabilization	NM_005652.2	5′-UTR (-20)	GGGAGGGCGGGGAGGG	Gomez et al. 2010 [69]	NM_001286200.1	65	1.00

A sampling of human G-quadruplexes reported in the published literature, along with their mouse QGRS orthologs mapped with QGRS-Conserve. (Many of these conserved motifs were also successfully identified in additional mammalian species; data not shown). ^a^Values obtained by semi-global alignments between human and mouse orthologous mRNAs. ^b^Relatively lower conservation score noted due to slight differences in the number of tetrads, loop lengths, and overall lengths of aligned motifs. However, sequence alignment showed high location conservation. ^c^Homologous QGRS mapped upstream of the mouse orthologous mRNA TSS.

Among the ten selected G-quadruplexes, the QGRS found in the KISS1 gene offer an example of where our method does not locate a highly conserved (conservation score ≥0.95) homologous QGRS. The G-quadruplex in the 3′-UTR of KISS1 was matched with a QGRS yielding a relatively lower conservation score of 0.706 (Table 2). Close examination of the KISS1 QGRS pair shows that the nearest mouse QGRS differs significantly by number of tetrads (2 vs 4), loop lengths (7, 3, 16 vs 8, 4, 3 nt) and overall length (42 vs 23 nt) when compared to the published human G-quadruplex [67]. The alignment between KISS1 human and mouse mRNA sequences is relatively low (49% overall similarity), particularly in the 3′-UTR; and it is difficult to determine if this particular human G-quadruplex is conserved. In this case, a known human G-quadruplex would likely be filtered out of search results if a user of our system was examining QGRS with only high conservation scores (ex., 0.95+). This is simply a characteristic of our approach - while conservation strongly suggests biological relevance, it is not necessarily a prerequisite for a QGRS to form a G-quadruplex in living cells - and users of our tools must recognize this important point.

## Conclusions

QGRS-Conserve can identify G-quadruplex-forming sequence motifs conserved among homologous nucleic acids. Identifying evolutionarily conserved motifs helps validate computations and provides evidence for their biological relevance. Ours is one of the first strategies for quantitatively evaluating conservation of G-quadruplex motifs (QGRS) using both the location as well as structural characteristics of motif pairs. Coupled with filtering approaches related to predicted stability [53,54], conservation scoring is very helpful in limiting false positives when identifying QGRS, especially for analyzing large datasets.

We have applied QGRS-Conserve to identify multiple homologous motifs of several previously published G-quadruplexes involved in regulating gene expression and human disease; however, we have only begun the analysis of exomic data across multiple species.

Our analysis has focused on a variety of information for categorization of QGRS including their structural characteristics, relative location, and mRNA characteristics. We have identified many homologous G-quadruplexes in the regulatory 5′- and 3′-untranslated regions and discovered significant differences in their distribution patterns among phylogenetic divisions. Our data is searchable through an interactive web application at http://qdb.ramapo.edu.

Looking at aggregate distribution patterns can be helpful for gaining insights into overall conservation of QGRS and their conserved biological roles. However, one of the most important benefits of QGRS-Conserve is the ability to quickly and efficiently identify conserved QGRS in regions where there is relatively little overall sequence conservation. QGRS regions that exhibit higher conservation than their general location in an mRNA offer exciting areas of study, as their conservation suggests regulatory roles. It will be important, as this research continues, to apply new computational methods to compare the QGRS conservation with the surrounding areas of the nucleic acid sequence to isolate regions with different evolutionary constraints and correlate it to their biological relevance.

General observations can be made when looking at entire transcriptomes/exomes; however, when nucleic acids are grouped by more specific characteristics (such as gene function), these analyses may yield more actionable results and help reveal information concerning the biological roles of the G-quadruplex in the genomes. Furthermore, studying the quantitative distribution of G-quadruplex motifs relative to functional hot spots on nucleic acids - such as polyA sites, translation initiation and termination sites, and splice sites - within a variety of mRNA clusters, can provide valuable insights into both the role of the G-quadruplexes and their relationships with the mRNAs themselves. The development of computational tools such as those presented allows us to build datasets that can be filtered and categorized in a variety of dimensions and allows these critical research questions to be better addressed.

## Abbreviations

ADAM10: A Disintegrin and Metallopeptidase Domain 10; APP: amyloid precursor protein; CDS: coding sequence; DNA: deoxyribose nucleic acid; EMBOSS: European Molecular Biology Open Software Suite; FGF2: fibroblast growth factor 2; IGF2: insulin-like growth factor 2; KISS1: Kisspeptin1; IRES: Internal Ribosomal Entry Site; LRP5: low-density lipoprotein receptor-related protein 5; MeCP2: methyl CpG binding protein-2; mRNA: messenger RNA; NCBI: National Center for Biotechnology Information; NRAS: neuroblastoma Ras; QGRS: quadruplex forming G-rich sequence(s); PIM1: Pim1 proto-oncogene; PSD95: postsynaptic density protein 95; RNA: ribonucleic Acid; SNCA: synuclein, alpha (non-A4 component of amyloid precursor); TERF2: telomeric repeat binding factor 2; TSS: transcription start site; UTR: untranslated region; VEGF: vascular endothelial growth factor; YY1: Ying Yang 1.

## Competing interests

The authors declare that they have no competing interests.

## Authors' contributions

CM and MC participated in the implementation of application code and performed extensive testing and validation on the software. SF designed the application architecture, including the design and implementation of all algorithms and the methods used to manage and present the collected data. PB conceived of this study and provided expertise and leadership in the development of the algorithms and methods from a biological perspective. All authors made significant contributions to the creation of this manuscript and have read and approved its final version.
